# Current Understanding of COVID-19 Clinical Course and Investigational Treatments

**DOI:** 10.3389/fmed.2020.555301

**Published:** 2020-10-21

**Authors:** Richard B. Aguilar, Patrick Hardigan, Bindu Mayi, Darby Sider, Jared Piotrkowski, Jinesh P. Mehta, Jenankan Dev, Yelenis Seijo, Antonio Lewis Camargo, Luis Andux, Kathleen Hagen, Marlow B. Hernandez

**Affiliations:** ^1^Cano Health, Miami, FL, United States; ^2^Dr. Kiran C. Patel College of Allopathic Medicine, Nova Southeastern University, Fort Lauderdale, FL, United States; ^3^Dr. Kiran C. Patel College of Osteopathic Medicine, Nova Southeastern University (NSU), Fort Lauderdale, FL, United States; ^4^Internal Medicine, Cleveland Clinic Florida, Weston, FL, United States; ^5^Cleveland Clinic Florida, Weston, FL, United States; ^6^Health Professions Division, Nova Southeastern University (NSU), Fort Lauderdale, FL, United States

**Keywords:** COVID 19, infectious disease, disease management, directed treatment, cover-19 testing, clinical course

## Abstract

**Importance:** Currently, there is no unified framework linking disease progression to established viral levels, clinical tests, inflammatory markers, and investigational treatment options.

**Objective:** It may take many weeks or months to establish a standard treatment approach. Given the growing morbidity and mortality with respect to COVID-19, this systemic review presents a treatment approach based on a thorough review of scholarly articles and clinical reports. Our focus is on staged progression, clinical algorithms, and individualized treatment.

**Evidence Review:** We followed the protocol for a quality review article proposed by Heyn et al. (1). A literature search was conducted to find all relevant studies related to COVID-19. The search was conducted between April 1, 2020, and April 13, 2020, using the following electronic databases: PubMed (1809 to present); Google Scholar (1900 to present); MEDLINE (1946 to present), CINAHL (1937 to present); and Embase (1980 to present). The keywords used included *COVID-19, 2019-nCov, SARS-CoV-2, SARS-CoV*, and *MERS-CoV*, with terms such as *efficacy, seroconversion, microbiology, pathophysiology, viral levels, inflammation, survivability*, and *treatment and pharmacology*. No language restriction was placed on the search. Reference lists were manually scanned for additional studies.

**Findings:** Of the articles found in the literature search, 70 were selected for inclusion in this study (67 cited in the body of the manuscript and 3 additional unique references in the Figures). The articles represent work from China, Japan, Taiwan, Vietnam, Rwanda, Israel, France, the United Kingdom, the Netherlands, Canada, and the United States. Most of the articles were cohort or case studies, but we also drew upon other information, including guidelines from hospitals and clinics instructing their staff on procedures to follow. In addition, we based some decisions on data collected by organizations such as the CDC, FDA, IHME, IDSA, and Worldometer. None of the case studies or cohort studies used a large number of participants. The largest group of participants numbered <500 and some case studies had fewer than 30 patients. However, the review of the literature revealed the need for individualized treatment protocols due to the variability of patient clinical presentation and survivability. A number of factors appear to influence mortality: the stage at which the patient first presented for care, pre-existing health conditions, age, and the viral load the patient carried.

**Conclusion and Relevance:** COVID-19 can be divided into three distinct stages, beginning at the time of infection (Stage I), sometimes progressing to pulmonary involvement (Stage II, with or without hypoxemia), and less frequently to systemic inflammation (Stage III). In addition to modeling the stages of disease progression along with diagnostic testing, we have also created a treatment algorithm that considers age, comorbidities, clinical presentation, and disease progression to suggest drug classes or treatment modalities. This paper presents the first evidence-based recommendations for individualized treatment for COVID-19.

## Highlights

-**Question:** What are the most effective treatment recommendations for COVID-19?
-**Findings:** COVID-19 can be divided into three distinct Stages, beginning at the time of infection (Stage I), sometimes progressing to pulmonary involvement (Stage II, with or without hypoxemia) and less frequently to systemic inflammation (Stage III). In addition to modeling the stages of disease progression, we also created a treatment algorithm which considers age, comorbidities, clinical presentation, and disease progression to suggest drug classes or treatment modalities.
-**Meaning:** This paper presents the first evidence-based recommendations for individualized treatment for COVID-19.

## Introduction

The coronavirus disease 2019 (COVID-19) pandemic has spread throughout the globe. According to the Centers for Disease Control and Prevention (CDC), in the United States alone there were 5,460,429 cases along with 171,012 deaths, as of August 19, 2020 (2). A mathematical model created by The Institute for Health Metrics and Evaluation (IHME) predicts that in the United States the number of deaths may climb to over 295,000 by December 1, 2020 (3). This creates a critical and immediate need for medical treatment and resources.

**Figure 1:**
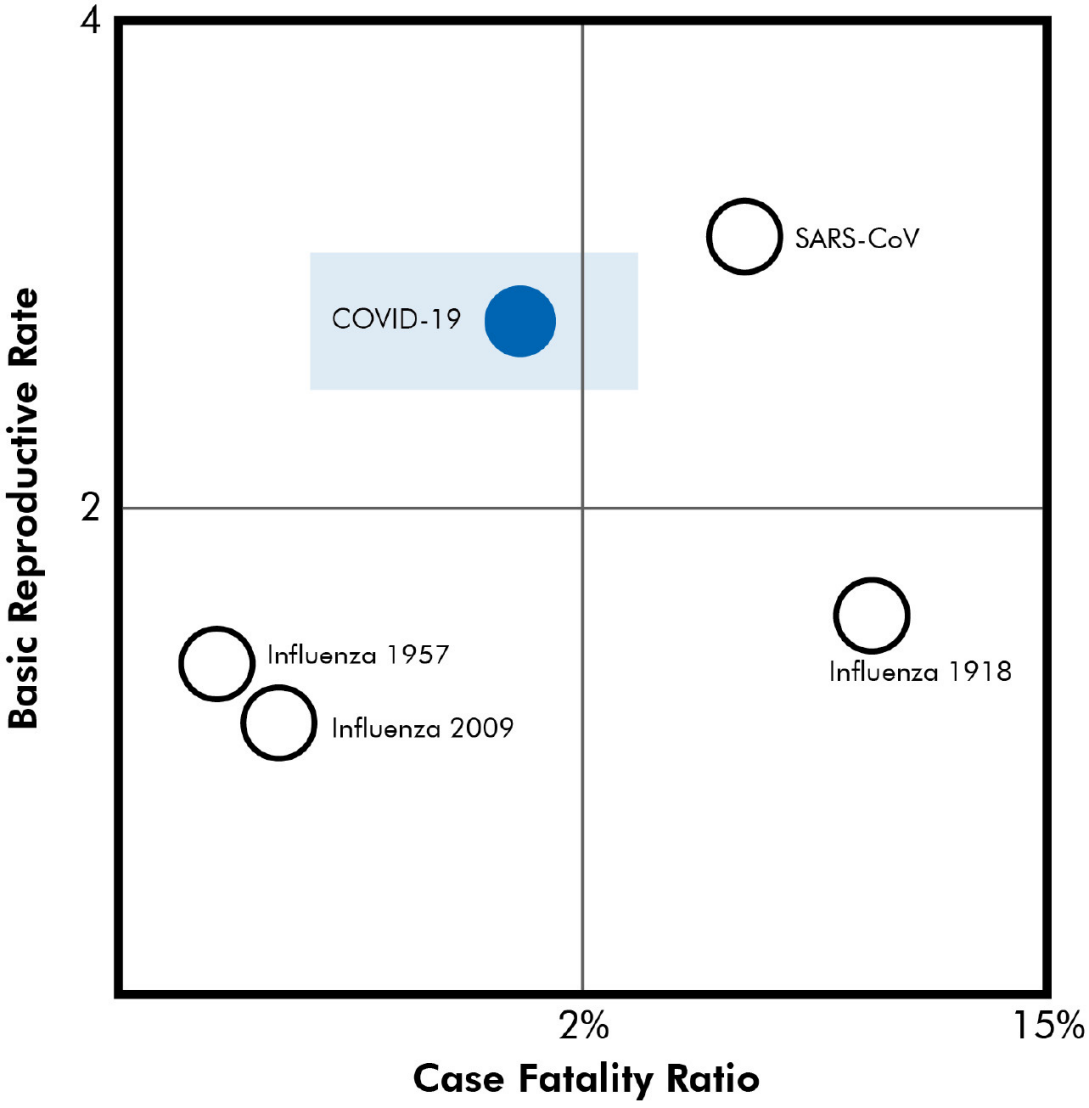
Reproduction rate and case-fatality rates for major respiratory virus pandemics (4, 5). Rectangle donates case fatality range from multiple publications.

Preliminary data in the US suggests that COVID-19 may be more infectious and lethal than Influenza H1N1. To place this in context, Figure 1 provides a comparison of the reproduction rate and case-fatality rates for major respiratory virus pandemics (4–6). In the general population, case-fatality rates for COVID-19 are about 1.4% (7). Data strongly emphasizes early intervention to reduce case-fatality and inhibit reproductive rates.

To date, a number of articles have been published on the clinical course and treatment of the disease (8–10). The majority of patients present with more than one symptom on admission, although the combination of fever, cough, and shortness of breath is rare. Siddiqi and Mehra proposed a staged progression model based on observed clinical courses in published studies (11). In Stage 1, or the mild phase, the virus multiplies and establishes residence in the host, predominantly in the respiratory tract. In Stage 2, there is viral multiplication and localized inflammation in the lungs. Stage 3 is marked by extra-pulmonary systemic hyperinflammation syndrome. The prognosis and recovery from Stage 3 is generally poor. Rapid recognition of which stage the patient is in and the deployment of appropriate therapy may have the greatest yield.

Common correlating factors that tend to lead to poorer outcomes include age, hypertension, diabetes, coronary artery disease, chronic lung disease, and malignancies (12). Research also finds variations in outcomes due to a dysregulated and exuberant immune response. Patients requiring intensive care have significantly higher levels of IL-6, CRP, ferritin, and D-Dimer. An important therapeutic modality may be to downregulate the cytokine storm, particularly in severe illness (13). The literature also suggests that disease progression can be predicted. During the severe acute respiratory syndrome (SARS) pandemic, a retrospective analysis revealed that 2-week cumulative case data could help estimate the total case numbers with accuracy—well before the date of the last reported case (14).

As we have found, there is no unified framework linking disease progression to established viral levels, clinical tests, inflammatory markers, and investigational treatment options. Given that it may take many weeks or months to establish a standard treatment approach and that rates of morbidity and mortality are increasing, we present an initial treatment approach based on a thorough review of currently available scholarly articles and clinical reports. Our focus is on staged progression, clinical algorithms, and individualized treatment.

## Methods

We followed the protocol for a quality review article proposed by Heyn et al. (1). A literature search was conducted to find all relevant studies related to COVID-19. The search was conducted between April 1, 2020, and April 13, 2020, using the following electronic databases: PubMed (1809 to present); Google Scholar (1900 to present); MEDLINE (1946 to present), CINAHL (1937 to present); and Embase (1980 to present). The keywords used in this search included *COVID-19, 2019-nCoV, SARS-CoV-2, SARS-CoV*, and *MERS-CoV*, with terms such as *efficacy, seroconversion, microbiology, pathophysiology, viral levels, inflammation, survivability*, and *treatment and pharmacology*. No language restriction was placed on the search. Reference lists were manually scanned for additional studies. From this systematic review, a model was created that incorporated clinical course, diagnostics, disease management, and treatment.

**Figure 2:**
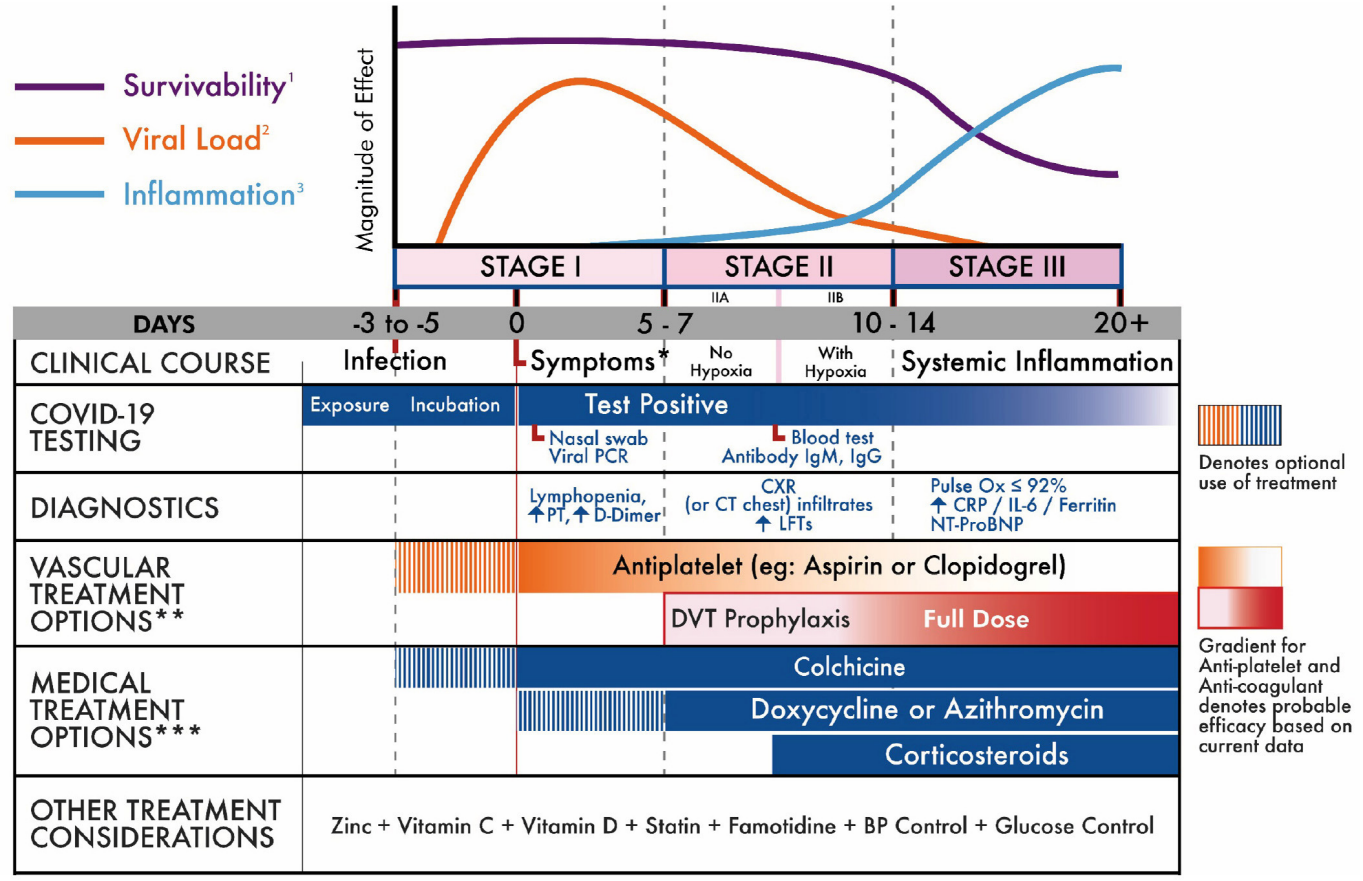
COVID-19 clinical stages and management strategy (15–17). *Initially mild in stage I (fever, cough, myalgia, other non-specific). May progress in stage II-III to severe dyspnea and respiratory distress (16, 18–20). **As with all treatment options, risks, and benefits should be carefully reviewed with the patient. ***No treatments are currently FDA approved for COVID-19 treatment. The FDA has approved remdesivir and convalescent plasma for inpatient use.

**Figure 3:**
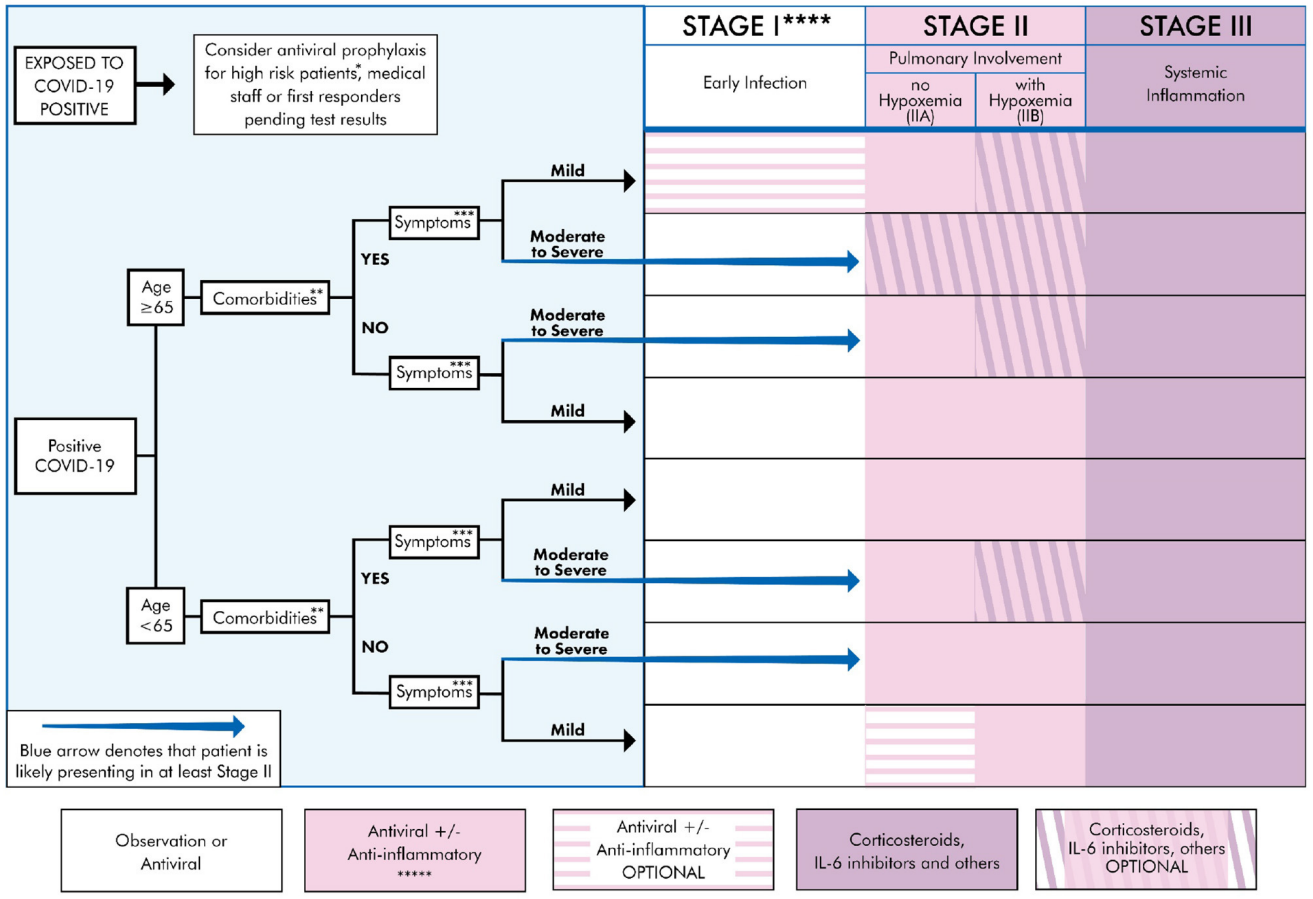
Treatment algorithm for COVID-19+ patients based on clinical presentation and therapeutic staging. *High risk patient: Anyone that is ≥65 y/o or meets comorbidities criteria as defined below. **Comorbidities: Defined as any two of the following: HTN, DM, CVD, CKD, Pre-existing lung disease, CHF, diabetes >7.6%, use of biologicals, HIV+, history of transplant, morbid obesity (BMI ≥ 40) (21, 22). ***Symptoms Mild: Fever, cough, fatigue, myalgia, headache, anosmia. Rarely, patients may also present with diarrhea, nausea, and vomiting (8, 21, 23). Moderate: Symptomatic viral pneumonia with possible hypoxemia (PaO2/FiO2 < 300). Confirmed by chest imaging (CXR or CT) which demonstrate bilateral infiltrates or ground glass opacities (21). Severe symptoms: Systemic (extra-pulmonary) hyperinflammation with one of the following: respiratory rate > 30 or SpO_2_ < 92% on room air (11, 17). Will also include abnormal chest imaging (CXR, CT scan, or lung ultrasound) characterized by bilateral opacities that are not primarily due to volume overload or lung collapse (partial or full). Echocardiogram can be used rule out of primary cardiac causes (24, 25). ****See Table 1 for appropriate Rx for stage. Treatment must be individualized to the patient by considering risks, benefits, and contraindications of the particular Rx. Note: there may be a potential for combining multiple agents if no drug interaction exists, as there are pleural mechanisms of actions. *****Convalescent plasma can be used during any stage, though likely more beneficial earlier in the disease course (63).

Our results focus on recommendations for individualized treatment, by selecting the most appropriate drug or modality for the patient, carefully weighing risks and benefits. Clinicians and patients should understand the staged progression of COVID-19 (Figure 2). As such, we present a treatment algorithm that recommends no treatment for some and specific treatment for others, depending on age, comorbidities, and symptom severity (Figure 3).

## Results

Based on our thorough review of the literature, we correlated the disease course to COVID-19 testing, diagnostic options, and treatment strategies (see Figure 2). COVID-19 can be divided into three distinct stages, beginning at the time of infection (Stage I), sometimes progressing to pulmonary involvement (Stage II, with or without hypoxemia), and less frequently to systemic inflammation (Stage III). We also created a treatment algorithm that considers age, comorbidities, clinical presentation, and disease progression to suggest drug classes or treatment modalities (see Figure 3). The specific treatments are summarized in Table 1 (15, 21–62, 64).

**Table 1:** Summary of investigational treatments by COVD-19 effect.

**Agent**	**Effect**	**Dosing**	**Stage**	**Mechanism**	**Commentary**
Remdesivir	AV	•200 mg IV × 1, followed by 100 mg qd for 5–10 days	I–III	•RNA polymerase inhibitor (26)	•Adverse effects include elevated ALT and AST, phlebitis, constipation, headache, nausea. •Theoretical risk of renal injury. •Should not be used in pregnancy due to lack of data. •Has limited drug-drug interactions (no significant CYP effect) •Clinical trials underway in the US, UK, and China •Showed efficacy in COVID-19 treatment (27)
Lopinavir/Ritonavir	AV	•200 mg/50 mg/capsule, 2 capsules PO bid for no more than 10–14 days	I–III	•Protease inhibitor (28)	•Nearly 14% of patients cannot complete a course due to GI side effects (29). •Ritonavir is a potent CYP3A4 inhibitor (interacting with Rx such as apixaban, tacrolimus, and amiodarone). •In rare cases, Lopinavir/Ritonavir can cause liver injury, pancreatitis, and cardiac toxicity. •Treatment in mostly Stage II–III patients was not found to be superior to standard of care. Subgroup analysis suggestive that earlier treatment (Stage I) might be beneficial (28)
Favipiravir	AV	•1,600 mg PO bid x1d, then 600 mg PO bid for up to 14 days	I–III	•Broad spectrum inhibitor of RNA-dependent RNA polymerase (30, 31)	•Increases liver function parameters (AST, ALT, and total bilirubin) •Testis toxicity and has a risk for teratogenicity and embryotoxicity •Was found to be superior to Lopinavir/Ritonavir in a small controlled study (32)
Umifenovir	AV	•200 mg q8h for up to 14 days	I–III	•S protein/ACE-2 membrane fusion inhibitor (33)	•Metabolism by CYP3A4. Caution with strong inhibitors or inducers. •Hypersensitivity risk increases in children under 2 years of age •Limited clinical evidence shows promise in COVID-19 (34)
Hydroxychloroquine	AV A-IN	•Stage I–II −400 mg PO bid for first day followed by 200 mg bid daily for 5 days (35, 36) •Stage III—May consider extending treatment (200 mg bid) for up to 14 days (35)	I–III	•AV: replication-neutralization of the pH cellular organelles for gene replication •A-IN: inhibition of macrophage activation and reducing release of tissue TNF-a, IL-1, IL-6 (37–39) •A-IN: interfere with lysosomal activity and autophagy, disrupt membrane stability, alter signaling, and transcriptional activity, which can then inhibit immune activation and cytokine production (38)	•Adverse effects include: rash, nausea, and diarrhea. GI symptoms can be mitigated by taking with water; use with caution in diabetic patients may cause hypoglycemia (40) •Increased risk of retinopathy with a recommended maximal daily dose of 5.0 mg/kg. Avoid if history of retinal disease, macular degeneration, or previous treatment with tamoxifen (41) •Caution in patient at risk for QT prolongation. EKG at baseline and following initiation is generally advised, particularly in critically ill patients. •Contraindicated with Epilepsy, Porphyria, G6PD, and Myasthenia Gravis •Not proven effective for Pre-Exposure/Post-Exposure prophylaxis. IDS recommends for patients hospitalized with Pneumonia, as part of a clinical trial (29) •No definitive evidence from randomized controlled trials that it is effective (29)
Chloroquine	AV A-IN	•Stage I–II −500 mg bid for 5 days •Stage III—may consider extending treatment for up to 10 days	I–III	•A-IN: decrease secretion and/or receptor expression of cytokines such as TNF-a (39, 42) •Interfere with lysosomal activity and autophagy, similar to Hydroxychrloroquine (38)	•Has greater adverse event profile than Hydroxychloroquine and possibly less efficacy. Most common symptoms include abdominal cramps, nausea, anorexia. •Can increase QTc and result in hematologic effects (including hemolysis with G6PD deficiency). Can cause retinal toxicity and hypoglycemia. •As with Hydroxychloroquine, additional clinical trial data is necessary to determine efficacy and safety in COVID-19 patients (43). •No definitive evidence from randomized controlled trials that it is effective
Ivermectin	AV	•45–64 kg: 9 mg orally single dose •65–84 kg: 12 mg single dose •85 kg or more: 0.15 mg/kg orally single dose	I–II	•Broad-spectrum antiviral activity *in vitro* through inhibition of nuclear import of host and RNA viral proteins (44)	•May consider prophylactic use of Ivermectin in patients on corticosteroids who have high risk of Strongyloides hyperinfection •Possible considerations as adjunct therapy or replacement when other agents contraindicated •Contraindicated in Pregnancy
Ribavirin	AV	•IV 500 mg each time, bid or tid, no more than 10 days	II–III	•Inhibits viral RNA dependent RNA polymerase	•Can cause birth defects or death in an unborn baby (45) •Hematologic toxicity is observed in dose-dependent fashion. •Caution when used with azathioprine or HIV/AIDS medicines •Ribavirin is likely not effective when used alone and must be used in combination with IFN-α or lopinavir/ritonavir
Convalescent plasma donor containing SARS-CoV-2–specific antibody (IgG)	AV A-IN	•200–250 mL of ABO-compatible convalescent plasma × 2 (achieving 400 mL in total) on the same day it was obtained from the donor	I–III	•Neutralizing activity against SARS-CoV-2	•Allergic transfusion reactions •Likely most beneficial in early disease course (e.g., Stage I or Stage II), as its mechanism of action is to neutralize viral particles (46)
Azithromycin	A-IN	•500 mg qd × 1, then 250 mg bid for 4 days	II–III	•Inhibits RNA-dependent protein synthesis •Multiple immunomodulatory effects (47)	•Previous studies have shown some efficacy against viruses such as Influenza, Ebola, RSV, and Rhinovirus (48) •May confer benefit when added to Hydroxychloroquine (36, 49) •Should be used if superimposed bacterial Pneumonia. •Does increase QT interval, especially when added to Hydroxychloroquine. An EKG is recommended prior to start (and EKG or telemetry monitoring while on Tx is recommended) (50)
Doxycycline and other Tetracyclines	A-IN	•200 mg qd × 1, then 100 mg qd for 4 days. May consider extending treatment for up to 14 days	II–III	•Downregulation of NFkB pathway as well as TNFa, IL-1B and IL-6 •Possible inhibition of RNA replication (51)	•May confer benefit when added to Hydroxychloroquine. •Should be used if superimposed bacterial Pneumonia
Prednisone Methylprednisolone Dexamethasone Hydrocortisone	CS	•40–60 mg prednisone PO or 30–60 mg methylprednisolone IV, or 5–10 mg dexamethasone IV qd for up to 7 days •50 mg hydrocortisone IV q6H until improvement in shock	II–III	•Multiple immunomodulatory effects, including suppression of PMN migration and reversal of increased capillary permeability (52)	•Should not be used in Stage I (unless another indication) as it may increase viral load (53) •Indicated for asthma or COPD exacerbation or any shock with a history of chronic steroid use in excess of 10 mg prednisone daily. Also used for multipressor (>2 pressor) shock. •Use in patients with hypoxemia may confer a mortality benefit. If ARDS, higher doses may be required (54, 55)
Tocilizumab and other IL-6 inhibitors	A-IN	•4–8 mg/kg IV (usually 400 mg) × 1 dose. If inadequate response, may repeat one time after 12 h (56)	IIb–III	•Inhibits inflammatory cytokine storm •Inhibits IL-6 and signal transduction of RNA viruses but not of DNA viruses (57)	•Side effects include upper respiratory tract infections, mild stomach cramps. •Black box warning for a risk of serious infections, including tuberculosis and other opportunistic infections. Patients treated with this medication should be tested for latent tuberculosis prior to discharge from the hospital •Caution in neutropenia or thrombocytopenia •May interact with cholesterol-lowering medications, seizure medications, heart rhythm medications •May be beneficial for use in Cytokine Activation Syndrome (58)
IFN-α and other Type 1 Interferons	AV A-IN	•5 million U or equivalent dose each time, 2 times/day for Vapor inhalation	IIb–III	•Interfere with viral replication •Slowdown of cell metabolism and secretion of cytokines	•Inhalation pharmacodynamics and pharmacokinetics have never been assessed. •IV and SC modes of administration are well-described and proven safe in several clinical trials (under expert use), with similar pharmacodynamics and pharmacokinetics (59)
Prazosin and other alpha-1 adrenergic receptor (AR) antagonists	A-IN	•1 mg bid or tid, titrating up as tolerated	I–III	•Reduces catecholamine and cytokine response through alpha-1 AR antagonism	•Contraindicated if hypotension. •No current evidence for starting the Rx if patient is not already on it. A recent retrospective review found that patients previously treated with alpha-1AR antagonists had improved end points (60)
Atorvastatin and other Statins	A-IN	•Atorvastatin 40 mg qhs	I–III	Pleiotropic effects, anti-inflammatory	•If there is an indication for a statin, the statin should be started or continued (15)
Baricitinib	A-IN AV?	•Eli Lilly and National Institute for Allergies and Infectious Diseases (NIAID) announced that the drug will begin its first large randomized trial in COVID-19 patients, in late April in the U.S., and additional sites in Asia/Europe (61)	IIb–III	•JAK1/JAK2 inhibitor •Theoretic (but proven) antiviral properties	•FDA approved for treatment of rheumatoid arthritis. •Side effects include upper respiratory tract infection and reactive of herpes simplex and herpes zoster. •Black box warning for serious infections including TB. Patients must be tested for TB prior to starting treatment. •Increase risk of malignancy (including Lymphoma), and thromboembolism (61)
Colchicine	A-IN	•1.5 mg loading dose + 0.5 mg after 60 min, and then 0.5 mg bid for up to 3 weeks (62)	I–II	•Anti-inflammatory, through a variety of mechanisms including inhibition of neutrophil chemotaxis and IL-1 activation	•FDA approved for the treatment of gout and familial Mediterranean fever •Most common adverse effects are abdominal pain and diarrhea. •Adequate cardiovascular safety profile
Heparin, Enoxaparin, and other Anticoagulants	AC	•DVT Prophylaxis Dosing (e.g., Enoxaparin 40 mg SC qd, or Heparin 5000 SC tid) •Full anticoagulation—individualize to patient	II–III	Tissue factor pathway inhibition (54)	•Indicated as DVT prophylaxis for all hospitalized patients (Stage II) without contraindication for anticoagulation. •Full anticoagulation may be beneficial in Stage III, as it has shown benefit for those suffering from sepsis associated coagulopathy, ARDS, or D-Dimer levels >6-fold the upper limit of normal (54)

*AV, Antiviral; A-IN, Anti-inflammatory; CS, Corticosteroid; AC, Anti-coagulant*.

* *None of these Rx are considered standard of care for treatment of COVID-19, and ideally should be used as part of a clinical trial. Moreover, this table is not meant to be a comprehensive review of adverse effects and drug-drug interactions. Treatment must be individualized to the patient, considering the patient's age, comorbidities, clinical course, drug interactions, and hypersensitivities. Lastly, this table is meant to be updated as new evidence (and perhaps new agents or classes of agents) is presented*.

### Comorbidity

Data exists for early identification of cases at high risk of progression to severe COVID-19. One promising model created in China found that patients who developed severe COVID-19 possessed one of the following diseases: hypertension, diabetes, coronary heart disease, chronic respiratory disease, or tuberculosis. The same model cited age and various serological indicators [such as C-reactive protein (CRP), lactate dehydrogenase (LDH), bilirubin, and others] as factors associated with worse outcomes (65). Additional research confirmed, in a case-control study, that subjects with high Sequential Organ Failure Assessment (SOFA) scores, with age >65, with hypertension, diabetes, and/or coronary heart disease were at greatest risk (66). Lastly, research focusing on viral load and survival found that higher initial viral load is independently associated with worse prognosis (2).

### Disease Progression

The most common presenting symptoms are fever and cough, followed by myalgia and fatigue. Less commonly, patients may present with sputum production, headache, or abdominal symptoms like diarrhea (21). In terms of disease progression, a case study of the first five patients diagnosed with COVID-19 in Europe points the way to two different clinical evolutions of the disease: 1. Presenting few symptoms, but showing high viral load from the respiratory tract; 2. A two-step disease process, with worsening of symptoms around 10 days of symptom onset despite decreased viral load in respiratory samples. In our model, we plot the disease progression as a function of infection, survivability, and inflammation (Figure 2).

**Figure 4:**
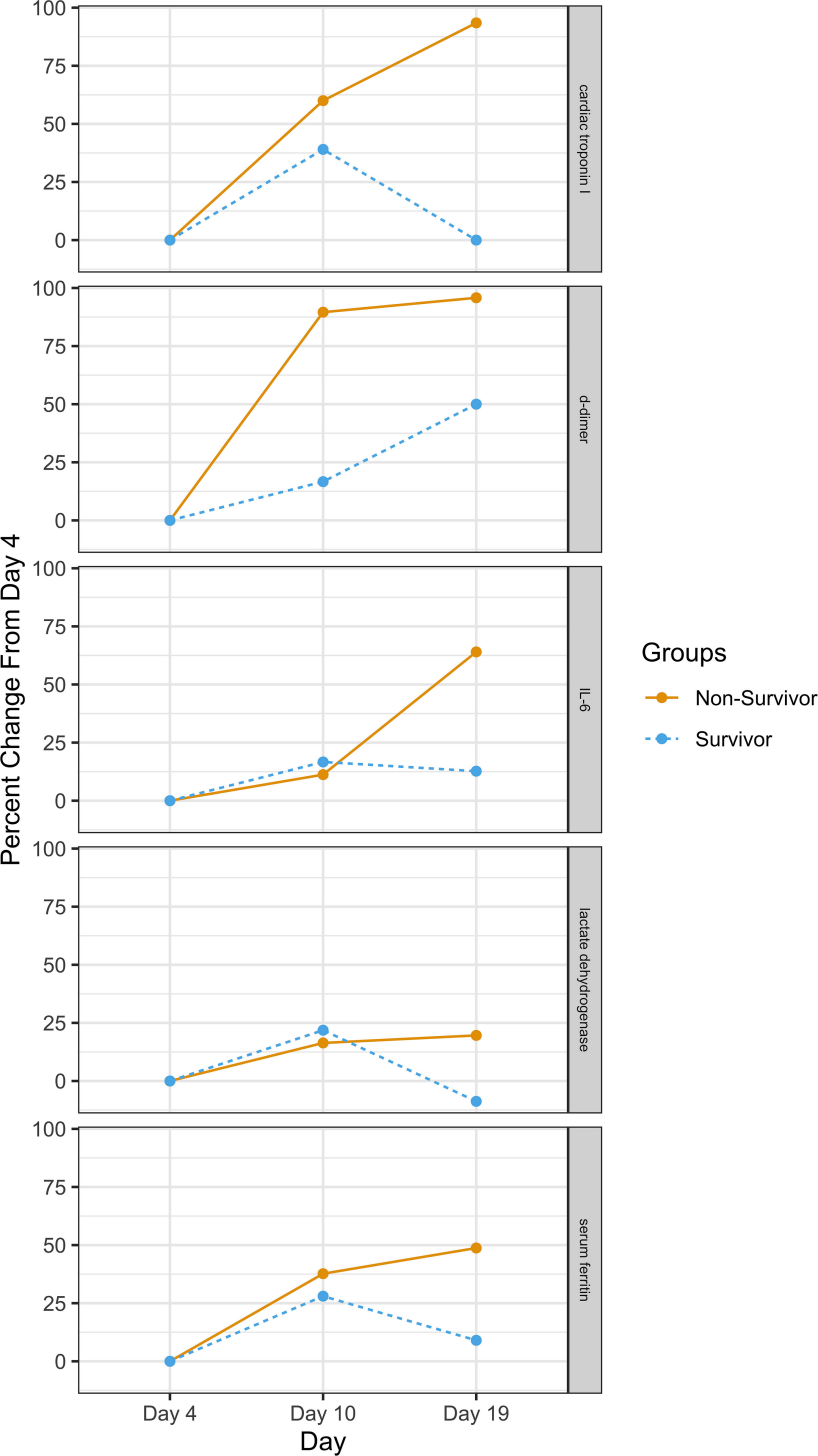
Percent change in clinical measures between survivors and non-survivors. Source: (21).

We identify the inflection point where survival decreases as inflammation increases—approximately day 10 from symptom onset. Support for this is found in research by Chen et al. published in *The Journal of Infection* (37). Their research found that sepsis and ARDS in hospitalized patients start at around days 10 and 11, respectively. They also found temporal changes in inflammatory laboratory markers beginning at day 4 of illness onset. These included temporal changes in D-dimer, IL-6, serum ferritin, high-sensitivity cardiac troponin I, and lactate dehydrogenase. The differences were statistically significant between survivors and non-survivors for all time points. Figure 4 provides the percent change between survivors and non-survivors from day 4. In addition, Yang et al. found that the patients admitted to the ICU with severe hypoxemia had a 50% probability of survival at day 7 of ICU admission (corresponding to Day ~17 in Figure 2) (16).

### Stage I

The incubation period is on average 5 days. In most patients, initial presenting symptoms are mild (though a small number of patients can be asymptomatic throughout the disease). Stage I symptoms include fever, cough, fatigue, and body aches. In a minority of cases, symptoms may also include headache, abdominal symptoms, anosmia, as well as others. The duration of initial symptoms is 5–7 days, correlating with a peak in viral load (21). During this time, the appropriate diagnostic test is a nasopharyngeal PCR. Laboratory studies may include an elevated D-Dimer and prothrombin time, as well as lymphopenia (see Figure 2). Given that symptoms in this stage are mild, and correlated with viremia, the appropriate treatment modality is supportive care or antiviral medication. Nevertheless, treatment must be individualized, based on a patient's age, comorbidities, presenting symptoms, and drug interactions (see Figure 3 and Table 1).

### Stage II

Some patients progress into Stage II, which is characterized by a decrease in viral levels and an increase in inflammation that initially localizes to the lungs. Infiltrates are typically seen on chest x-ray (CXR) or computed tomography (CT). Similar to symptom duration in Stage I, the typical symptom course in Stage II is also 5–7 days. Treatment with antivirals is still indicated, but given an average decrease in viral levels during this stage, that treatment is theoretically less effective than in Stage I. Moreover, Stage II is divided into two sub-stages (IIA and IIB), depending on whether a patient is hypoxemic or not. This distinction is important for management (see Figure 2). In Stage IIB, the patient is significantly dyspneic and may benefit, depending on age and comorbidities, from the use of corticosteroids or other anti-inflammatory treatments (see Figure 3).

### Stage III

Although only a minority of patients (estimated at 10–15%) progress to Stage III, mortality within this stage is considerable (estimated at 20–30%). The morbidity and mortality are generally due to uncontrolled inflammation, which at this point is systemic. The most important symptom is respiratory distress (correlating in a typical patient to a Pulse Ox ≤ 92%). Laboratory markers include significantly increased CRP and IL-6 levels (16, 67). As in Stage II, treatment may include antivirals (if the patient is still viremic), but agents to counteract inflammation and its effects (such as microthrombi) must be considered (see Figure 2). A summary of investigational therapies can be found in Table 1. It should be noted that many ongoing clinical trials will more clearly define COVID-19 specific treatment risks and benefits.

### Pre-exposure and Post-exposure Prophylaxis

A number of clinical trials are exploring pre-exposure and post-exposure prophylaxis. There is no definitive evidence that any particular treatment modality is effective but antivirals, anti-parasitics, and convalescent plasma have been proposed. Antivirals, like Remdesivir, may prove beneficial at any stage of disease (26, 27). Convalescent plasma provides the antibody support needed to envelop and destroy the virus while preventing the exuberant immune response or cytokine release that leads to significant pathology, particularly in Stages IIb and III (46).

## Limitations

This review has several limitations. First, the incredible volume and speed at which data is published about the treatment of COVID-19 indicates that research findings and recommendations may change. Second, the research used to create this review came from small studies, often-times with very few controls. Third, the articles were limited to English-language publications or translations, so relevant international data could be lacking.

## Conclusion

This paper presents the first evidence-based recommendations for individualized treatment for COVID-19. Based upon the observed transmission and mortality rates, health professionals urgently need to align patient baseline risk to disease stage and investigational treatment options. The COVID-19 pandemic represents the greatest public health crisis in three generations: the need for comprehensive management cannot be overstated.

## Data Availability Statement

The original contributions presented in the study are included in the article/supplementary material, further inquiries can be directed to the corresponding author.

## Author Contributions

MH and RA revised the project, the main conceptual ideas and proof outline. BM, DS, JP, JPM, JD, YS, AC, and LA worked out almost all of the technical details with MH and RA assistance. PH, KH performed the numerical calculations and verified the numerical results. All authors contributed to writing and editing the manuscript.

## Conflict of Interest

The authors declare that the research was conducted in the absence of any commercial or financial relationships that could be construed as a potential conflict of interest.
